# Long-term visual acuity outcome of pediatric uveitis patients presenting with severe visual impairment

**DOI:** 10.1038/s41598-023-29159-x

**Published:** 2023-02-20

**Authors:** Usanee Tungsattayathitthan, Narisa Rattanalert, Wantanee Sittivarakul

**Affiliations:** Department of Ophthalmology, Faculty of Medicine, Prince of Songkla University, 15 Karnjanavanich Road, Hat Yai, 90110 Songkhla Thailand

**Keywords:** Paediatric research, Immunology

## Abstract

This study investigated the long-term visual acuity (VA) outcome in the eyes of children with uveitis and severe visual impairment (SVI; VA ≤ 20/200) at presentation. Fifty-one children [57 eyes; median age, 11 years; 51% female; median follow-up period, 36 months (interquartile range 14.9–64.4)] aged ≤ 16 years with uveitis managed at our tertiary center from January 2010 to July 2020 were reviewed. Uveitis mainly manifested as unilateral (74.5%), chronic course (82.4%), and panuveitis (43.1%). Ocular toxoplasmosis and toxocariasis were the most common diagnoses (9.8% each). At least one ocular complication at presentation was observed in 93% of the eyes. Overall, the mean logMAR VA improved from 1.8 at presentation to 1.2 at 5 years (P < 0.001). Common causes of poor vision included retinal detachment, atrophic bulbi, and optic atrophy. Predictive factors associated with less VA improvement over the follow-up period included preschool age of uveitis onset (P < 0.001), ocular symptoms duration before uveitis diagnosis ≥ 1 month (P = 0.004), and non-anterior uveitis (P = 0.047). The long-term VA outcome in uveitis-affected eyes with SVI at presentation was unfavorable. Younger age at uveitis onset, delayed presentation, and uveitis involving the posterior segment were associated with poorer VA outcome.

## Introduction

Uveitis in the pediatric population is one of the most complex ophthalmic conditions associated with high ocular morbidity. The diagnosis and management of these children often present specific challenges and require specialist care, given the potential for delayed diagnosis, difficulty in thorough ocular examination, and risk of amblyopia^1^. The incidence of pediatric uveitis is estimated to be 4.3 to 14 per 100,000 person-years^2,3^. Clinic-based surveys demonstrated that children accounted for only 2.2–13.8% of all uveitis patients presenting to tertiary clinics^1,4,5^. However, severe visual acuity (VA) loss at presentation has been reported to range from 6.4 to 45% among this group^6–13^. This high rate of visual loss is a serious concern because of the greater life span of children compared to that of adults. Furthermore, long-term visual disability could affect several aspects of children’s lives, including physical, social, emotional, and cognitive development, resulting in poor quality of life^14^.

Our previous study demonstrated that approximately 40% of eyes of children with uveitis suffered severe visual impairment (SVI), defined as VA ≤ 20/200, at first presentation. Additionally, SVI at presentation was the only predictive factor of poor VA outcome at 1 year after presentation to our tertiary center^12^. However, the long-term pattern of VA outcome in pediatric patients with uveitis who presented initially with SVI is not well characterized. Whether these children could experience recovery of VA and whether any presenting clinical characteristics could predict VA improvement following uveitis subspecialty care management remain unclear. To address these questions, we analyzed the longitudinal pattern of VA as well as the predictive factors associated with VA improvement in a cohort of children with uveitis who had SVI at initial presentation in at least one eye managed at our tertiary center.

## Methods

### Study population

All new consecutive pediatric patients aged 16 years or less with uveitis who presented with VA ≤ 20/200 in at least one eye and were managed at Songklanagarind Hospital, a tertiary referral center in Southern Thailand, between January 2010 and July 2020 were included in this retrospective study. Patients diagnosed with exogenous endophthalmitis, trauma-induced uveitis, or lens-induced uveitis were excluded. Children with a follow-up period of less than 6 months, who could not undergo VA assessment, or who did not have VA data for the first visit were also excluded. Details of the study population have been described in our previous study^12^. The Ethics Committee of the Faculty of Medicine, Prince of Songkla University approved this study (no. 64-127-2-1) and waived the requirement for written patient informed consent as this research posed less than minimal risk to patients and because the rights and welfare of the patients would not be adversely affected by this study. The patient data were maintained confidentially and in compliance with the tenets of the Declaration of Helsinki.

### Data collection

Data on demographic characteristics, medical and ophthalmic histories, ophthalmologic examination at presentation, specific uveitis diagnosis, medication, and surgical treatment were collected. Age at uveitis onset was categorized into three groups: preschool (< 7 years), primary school (7–12 years), and secondary school (> 12–16 years). The anatomical location and course of uveitis were categorized according to The Standardization of Uveitis Nomenclature criteria^15^. All patients underwent a complete eye examination and additional systemic and ocular diagnostic testing as indicated by their clinical presentation. Diagnosis of each specific uveitic entity was based on standard diagnostic criteria^16–21^. Cases that did not show an association with any systemic or infectious disease or specific uveitis diagnosis were labeled as undifferentiated uveitis^22^.

The use of corticosteroids, immunomodulatory therapy (IMT), and biologic agents was managed according to the best medical judgment using published guidelines^23^. Specific anti-infectious medications were prescribed according to the causative pathogen. Surgical procedures included cataract surgery, glaucoma filtering surgery, and pars plana vitrectomy (PPV). The frequency of ocular complications observed at presentation and during follow-up was also assessed. Complications included cataract (grade ≥ 1 +), elevated intraocular pressure (IOP; > 21 mmHg), hypotony (IOP < 5 mmHg), posterior synechia, band keratopathy, retinal detachment (RD), optic disc hyperemia/edema, optic atrophy, neovascularization, vitreous hemorrhage, atrophic bulbi, macular scar/atrophy, macular edema, and epiretinal membrane.

VA was measured using the Early Treatment Diabetic Retinopathy Study chart. For young children who could not read numbers, preferential looking tests (Teller Acuity Cards^®^, Cardiff Acuity Test^®^) and picture charts were used as appropriate. VA data were collected at baseline; 3, 6, 12, 18, 24, 36, 48, and 60 months; and at the final visit. If there was no visit at these specified timepoints, the VA at a visit within 4–8 weeks of the timepoint was used. VA was converted to the logarithm of the minimum angle of resolution (logMAR) scale for statistical analysis. Children with VA of counting fingers, hand motion, light perception, and no light perception were assigned logMAR values of 2.2, 2.3, 2.4, and 2.5, respectively^24^.

### Outcome measure

The main outcome measure was the mean VA of the affected eye at the specified timepoints over the 5 years after presentation. For overall VA analyses, VA values were categorized into two groups based on uveitis diagnoses: (1) non-infectious uveitis (including undifferentiated uveitis) and (2) infectious uveitis. Secondary outcome measures were predictive factors associated with changes in VA over the 5-year follow-up period.

### Statistical analyses

Baseline characteristics are presented in terms of the frequency (%), mean and standard deviation (SD), median and interquartile range (IQR), or range, as appropriate. The Chi-squared test or Fisher’s exact test was used to analyze the relationships between categorical variables. The Mann–Whitney *U* test or independent *t* test was used to analyze the relationship between continuous variables. Longitudinal patterns of mean VA values during follow-up were analyzed using a mixed-effects linear model, with the patient and eyes considered as random elements and the interaction of time with the group as the fixed effect. Predictive factors associated with VA outcome were analyzed using univariate and multivariate linear mixed-effect regression models. As primary data inspection did not identify the differences in predicted VA over follow-up time between the non-infectious uveitis and infectious uveitis eyes, data of both groups were combined and analyzed together in the linear mixed-effect regression model to identify the predictive factors of VA outcome. Variables with a P-value < 0.2 were included in the initial multivariate model. The model was then refined by backward elimination of variables that did not contribute significantly to its fit, guided by the change in the log-likelihood of successive hierarchical models, retaining only the variables with P-value < 0.05. All statistical analyses were performed using STATA version 14 (StataCorp LP, College Station, TX).

## Results

### Demographic and clinical characteristics

A total of 123 patients with uveitis aged 16 years or younger were initially identified from our electronic database. Of these, 52 children presented with an initial VA ≤ 20/200 in at least one eye. One child was excluded because of less than 6 months of follow-up. Therefore, 51 patients (57 eyes) were included in the study. The median follow-up was 35.9 months (IQR 14.9–64.4), with 87.7%, 57.9%, and 45.6% of the eyes having at least 1-, 2-, and 3-year follow-ups, respectively.

Table 1 presents the demographic and clinical characteristics of the 51 patients categorized according to uveitis diagnosis. The median age at uveitis diagnosis in all patients was 9 years (IQR 6–11) and that at presentation to our clinic was 10.5 years (IQR 7.4–12.1). The median duration of ocular symptoms before uveitis diagnosis was 60 days (IQR 21–135). The majority of patients were female (51%), unilateral (74.5%), and had chronic uveitis (82.4%). Children with infectious uveitis were more likely to have posterior uveitis than those with non-infectious uveitis (P < 0.001). The mean logMAR VA of all affected eyes at initial presentation was 1.80 ± 0.57 (Snellen equivalent = 20/1262), and the mean logMAR VA at final follow-up was 1.26 ± 0.92 (Snellen equivalent = 20/364). Of the 51 children, 40 (78.4%) had VA in the better-seeing eye of > 20/50. Six children (11.8%) had bilateral SVI due to uveitis at presentation.

**Table 1:** Demographic and clinical characteristics of all 51 children (57 eyes) with uveitis presenting with severe visual impairment.

Characteristics	Total n = 51	Non-infectious n = 33 (64.7%)	Infectious n = 18 (35.3%)	P-value^a^
Age at presentation (years) (median, [IQR])	10.5 (7.4, 12.1)	10.5 (7, 11.4)	11 (7.9, 12.6)	0.608
Age group at onset, n (%)				
Preschool (< 7 years)	14 (27.5)	11 (33.3)	3 (16.7)	0.386
Primary school (7–12 years)	31 (60.8)	19 (57.6)	12 (66.7)	
Secondary school (> 12 years)	6 (11.8)	3 (9.1)	3 (16.7)	
Sex, n (%)				
Male	25 (49)	15 (45.5)	10 (55.6)	0.49
Female	26 (51)	18 (54.5)	8 (44.4)	
Course of uveitis, n (%)				
Acute	8 (15.7)	3 (9.1)	5 (27.8)	0.061
Chronic	42 (82.4)	30 (90.9)	12 (66.7)	
Recurrent	1 (2)	0	1 (5.6)	
Anatomical location, n (%)				
Anterior uveitis	3 (5.9)	3 (9.1)	0	< 0.001
Intermediate uveitis	10 (19.6)	10 (30.3)	0	
Posterior uveitis	16 (31.4)	3 (9.1)	13 (72.2)	
Panuveitis	22 (43.1)	17 (51.5)	5 (27.8)	
Type of uveitis, n (%)				
Granulomatous	7 (13.7)	5 (15.2)	2 (11.1)	1
Non-granulomatous	44 (86.3)	28 (84.8)	16 (88.9)	
Initial VA, n (%)				
20/200 to 5/200	26 (45.6)	17 (44.7)	9 (47.4)	0.347
< 5/200 to better than CF	1 (1.8)	0 (0)	1 (5.3)	
CF	7 (12.3)	6 (15.8)	1 (5.3)	
HM	15 (26.3)	11 (29.0)	4 (21.0)	
PL to No PL	8 (14.0)	4 (10.5)	4 (21.0)	
Initial VA, logMAR (mean, [SD])^b^	1.8 (0.6)	1.8 (0.6)	1.8 (0.6)	0.833
Final VA, logMAR (mean, [SD])^b^	1.3 (0.9)	1.3 (0.9)	1.25 (1)	0.976

*CF* counting fingers, *HM* hand motion, *IQR* interquartile range, *logMAR* logarithm of the minimum angle of resolution, *PL* perception of light, *SD* standard deviation, *VA* visual acuity.

^a^P-value for the comparison between the non-infectious and infectious groups.

^b^Analysis of a total of 57 uveitis affected eyes (38 eyes in the non-infectious group and 19 eyes in the infectious group).

Table 2 summarizes the uveitis diagnosis of all 51 patients. A specific diagnosis of uveitis was established in 24 (47.1%) patients. The remaining 27 (52.9%) children were considered to have undifferentiated uveitis. Uveitis of infectious etiology (35.3%) was roughly three times more common than immune-mediated uveitis (11.8%). Overall, the most frequent specific diagnoses were ocular toxoplasmosis and ocular toxocariasis (9.8% each), followed by diffuse unilateral subacute neuroretinitis (7.8%). Behçet’s disease was the most frequent diagnosis among children with immune-mediated uveitis (3.9%), followed by Vogt–Koyanagi–Harada (VKH) disease (2%), sarcoidosis (2%), systemic lupus erythematosus-related retinal vasculitis (2%), and pars planitis (2%).

**Table 2:** Diagnoses of all 51 children with uveitis.

Diagnosis	Number of patients (%)
Immune-mediated uveitis	6 (11.8)
Behçet’s disease	2 (3.9)
VKH	1 (2)
Sarcoidosis	1 (2)
SLE related retinal vasculitis	1 (2)
Pars planitis	1 (2)
Infectious uveitis	18 (35.3)
Ocular toxocariasis	5 (9.8)
Ocular toxoplasmosis	5 (9.8)
Diffuse unilateral subacute neuroretinitis	4 (7.8)
CMV retinitis	2 (3.9)
Necrotizing herpetic retinopathy	1 (2)
Tuberculous uveitis	1 (2)
Undifferentiated uveitis	27 (52.9)

*CMV* cytomegalovirus, *SLE* systemic lupus erythematosus, *VKH* Vogt–Koyanagi–Harada disease.

#### Treatment

Topical and systemic corticosteroids were prescribed to the majority of the children during treatment (84.3% and 80.4%, respectively). IMT was prescribed at some point during the treatment course to 18 of the 33 (54.5%) children with non-infectious uveitis; methotrexate monotherapy was the most frequently prescribed IMT (66.7%), whereas six children (33.3%) required combined IMT to achieve uveitis control. A biologic agent (infliximab) was prescribed to only one patient with Behçet’s disease.

One or more surgical procedures were performed in 31 eyes (54.4%) of 27 patients. PPV was the most frequently performed surgery (31.6%), followed by cataract surgery (24.6%) and glaucoma filtering surgery (10.5%). RD and epiretinal membrane were the most frequent indications for PPV.

#### Frequency of ocular complications at presentation and during follow-up

At presentation, at least one ocular complication was noted in 53 eyes (93%) of 48 children (Table 3). During follow-up, 40 eyes (70.2%) of 36 children developed new ocular complications that did not exist at presentation. The median number of complications per eye at any time during follow-up was 4 (range, 0–9). Among the three children (4 eyes) who did not have ocular complications at presentation, one child with undifferentiated intermediate uveitis developed cataract, IOP elevation, and macular edema during follow-up; the other two had no subsequent ocular complications.

**Table 3:** Ocular complications at presentation and during follow-up.

Complications	At presentation n (%)	During follow-up n (%)	Duration from presentation to complication onset in months (median [IQR])
Cataract	15 (26.3)	9 (15.8)	12.68 (7.68, 13.01)
Elevated IOP requiring management	6 (10.5)	19 (33.3)	4.51 (1.28, 12.09)
Posterior synechia	20 (35.1)	3 (5.3)	11.47 (11.47, 11.47)
Band keratopathy	18 (31.6)	8 (14)	13.75 (9.72, 27.81)
Retinal detachment			
Rhegmatogenous (RRD)	3 (5.3)	4 (7)	6.85 (5.49, 9.41)
Exudative (ERD)	4 (7)	0	–
Tractional (TRD)	4 (7)	9 (15.8)	11.73 (8.54, 31.80)
Optic disc hyperemia/edema	14 (24.6)	1 (1.8)	2.07 (2.07, 2.07)
Macular edema	14 (24.6)	4 (7)	14.57 (9.43, 41.33)
Macular scar/atrophy	2 (3.5)	1 (1.8)	14.55 (14.55, 14.55)
Epiretinal membrane	3 (5.3)	5 (8.8)	8.08 (4.53, 12.16)
Hypotony	4 (7)	6 (10.5)	7.80 (3.68, 48.36)
Optic atrophy	2 (3.5)	8 (14)	15.26 (5.09, 33.18)
Optic disc neovascularization	3 (5.3)	2 (3.5)	2.05 (0.46, 3.65)
Retinal neovascularization	3 (5.3)	2 (3.5)	3.94 (0.98, 6.90)
Iris neovascularization	1 (1.8)	5 (8.8)	35.91 (1.77, 59.92)
Vitreous hemorrhage	1 (1.8)	6 (10.5)	9.66 (1.15, 42.15)
Atrophic bulbi	1 (1.8)	7 (12.3)	16.98 (4.51, 40.93)
Central retinal vein occlusion	1 (1.8)	0	–

*ERD* exudative retinal detachment, *IOP* intraocular pressure, *IQR* interquartile range, *RRD* rhegmatogenous retinal detachment, *TRD* tractional retinal detachment.

#### VA outcome

**Figure 1:**
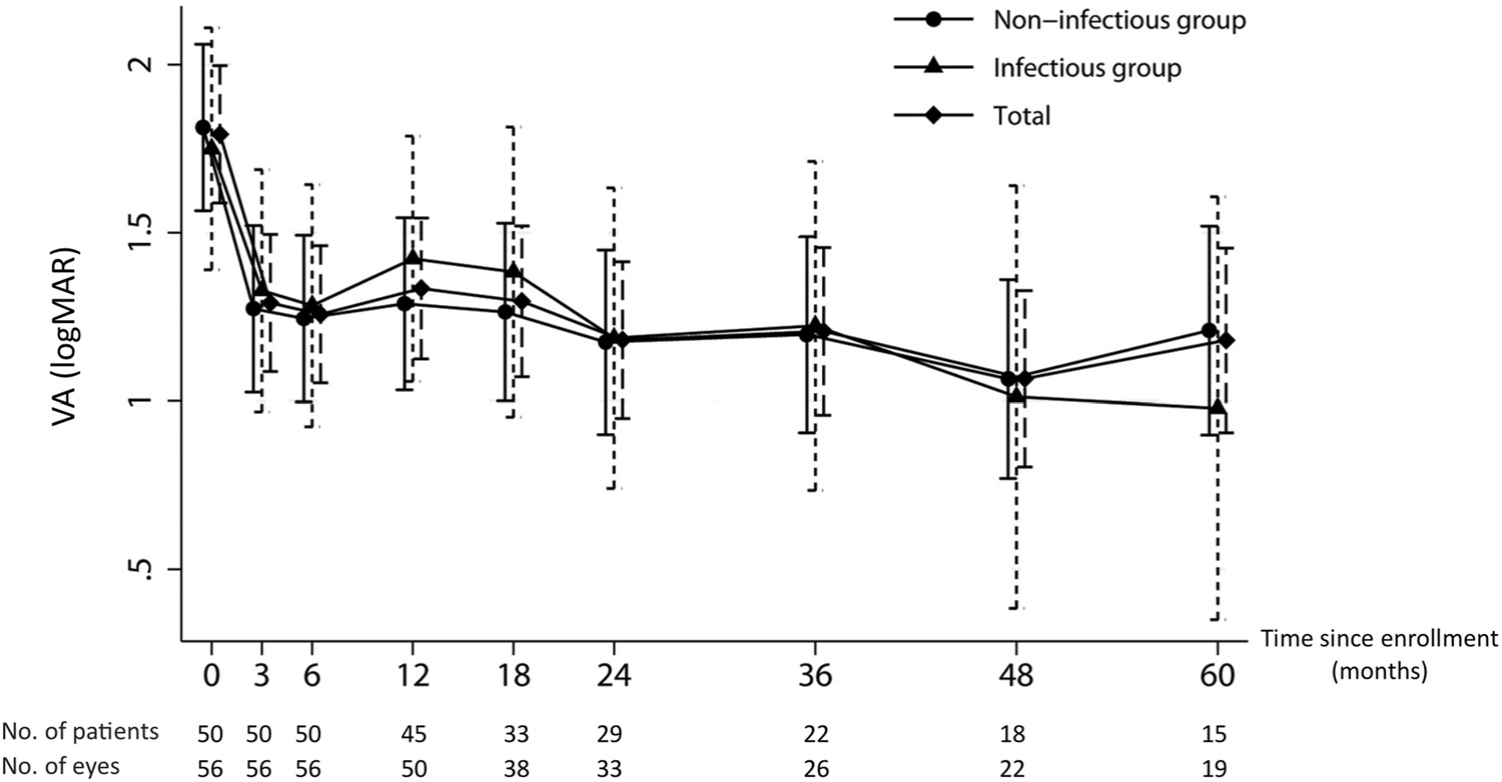
Estimated mean visual acuity (VA) over 5 years in all eyes and in eyes categorized into the non-infectious and infectious uveitis groups. Vertical lines represent 95% confidence intervals. *logMAR* logarithm of the minimum angle of resolution.

Figure 1 shows the mean VA of all 57 affected eyes as well as of eyes categorized into the non-infectious and infectious uveitis groups over the 5-year follow-up period, with the analyses performed using mixed-effects random intercept linear regression. Non-infectious uveitis eyes (mean logMAR VA = 1.81, [95% CI, 1.57–2.06], Snellen equivalent = 20/1291) and infectious uveitis eyes (mean logMAR VA = 1.75, [95% CI, 1.39–2.11], Snellen equivalent = 20/1125) both presented with relatively similar initial VA and showed an improvement of VA to 1.27 (Snellen equivalent = 20/372, P < 0.001) and 1.33 (Snellen equivalent = 20/428, P = 0.006), respectively, at 3 months. Thereafter, the mean VA appeared to be stably maintained throughout the 5-year follow-up. VA was relatively more stable in eyes with non-infectious uveitis than in eyes with infectious uveitis; however, no significant differences in VA at each timepoint were found between the non-infectious and infectious uveitis groups. At 5 years, the mean logMAR VA among eyes with non-infectious uveitis and those with infectious uveitis was 1.21 (Snellen equivalent = 20/324) and 0.97 (Snellen equivalent = 20/187), respectively, and was significantly more favorable than the VA at presentation (P < 0.001 and P = 0.011, respectively) (Table 4).

**Table 4:** Estimated mean logMAR VA of all eyes and in eyes categorized into the non-infectious and infectious uveitis groups.

	Baseline	3 months	6 months	12 months	18 months	24 months	36 months	48 months	60 months
Patients, n	50	50	50	45	33	29	22	18	15
Eyes, n	56	56	56	50	38	33	26	22	19
Mean logMAR VA of all eyes (95% CI)^a^	1.79 (1.59–2.00)	1.29 (1.09–1.50)	1.26 (1.05–1.46)	1.33 (1.12–1.54)	1.30 (1.07–1.52)	1.18 (0.95–1.41)	1.21 (0.96–1.46)	1.07 (0.80–1.33)	1.18 (0.91–1.45)
Mean logMAR VA of eyes with non-infectious uveitis (95% CI)^a^, n = 38 eyes	1.81 (1.57–2.06)	1.27 (1.03–1.52)	1.24 (1.00–1.49)	1.29 (1.03–1.54)	1.26 (1.00–1.53)	1.17 (0.90–1.45)	1.20 (0.91–1.49)	1.06 (0.77–1.36)	1.21 (0.90–1.52)
Mean logMAR VA of eyes with infectious uveitis (95% CI)^a^, n = 19 eyes	1.75 (1.39–2.11)	1.33 (0.97–1.69)	1.28 (0.92–1.64)	1.42 (1.06–1.79)	1.38 (0.95–1.81)	1.18 (0.74–1.63)	1.22 (0.74–1.71)	1.01 (0.38–1.64)	0.98 (0.35–1.61)
P-value^b^	0.777	0.808	0.862	0.555	0.643	0.964	0.928	0.880	0.518

*CI* confidence interval, *logMAR* logarithm of the minimum angle of resolution, *VA* visual acuity.

^**a**^Derived from mixed-effects random intercept linear regression model.

^b^P-value of Wald test comparing VA at each timepoint between the non-infectious and infectious uveitis groups.

**Figure 2:**
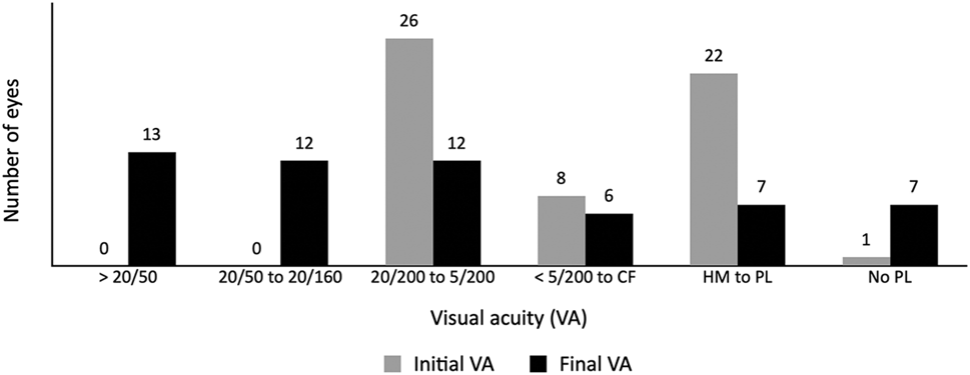
VA of included eyes at the initial and final visits. *CF* counting fingers, *HM* hand motion, *PL* perception of light, *VA* visual acuity.

By the time of the last follow-up, 13 of 57 eyes (22.8%) had achieved VA improvement to > 20/50, 12 eyes (21.1%) had achieved VA improvement to 20/50–20/160, and 32 eyes (56.1%) had persistent VA ≤ 20/200 (Fig. 2). The primary causes of SVI in these 32 eyes were tractional RD (10 eyes), atrophic bulbi (four eyes), rhegmatogenous RD (four eyes), optic atrophy (six eyes), cataract (three eyes), macular scar (one eye), glaucoma (three eyes), and band keratopathy (one eye). Forty-four of the 51 children (86.3%) were able to maintain VA in the better-seeing eye of > 20/50. Of these 44 children, 40 already had VA in the better-seeing eye of > 20/50 at presentation and their VA was maintained within this level throughout the final follow-up. The other four patients presented with a VA in the better-seeing eye of < 20/50; however, after treatment, their VA improved to > 20/50 at the final follow-up. Among the six children with bilateral uveitis who initially presented with bilateral SVI, VA of the better-seeing eye of three improved to 20/20, 20/25, and 20/80. The other three children (5.9%) had persistent SVI in both eyes and ended up with legal blindness (one with VKH disease and two with undifferentiated panuveitis).

#### Predictive factors of VA outcome

**Figure 3:**
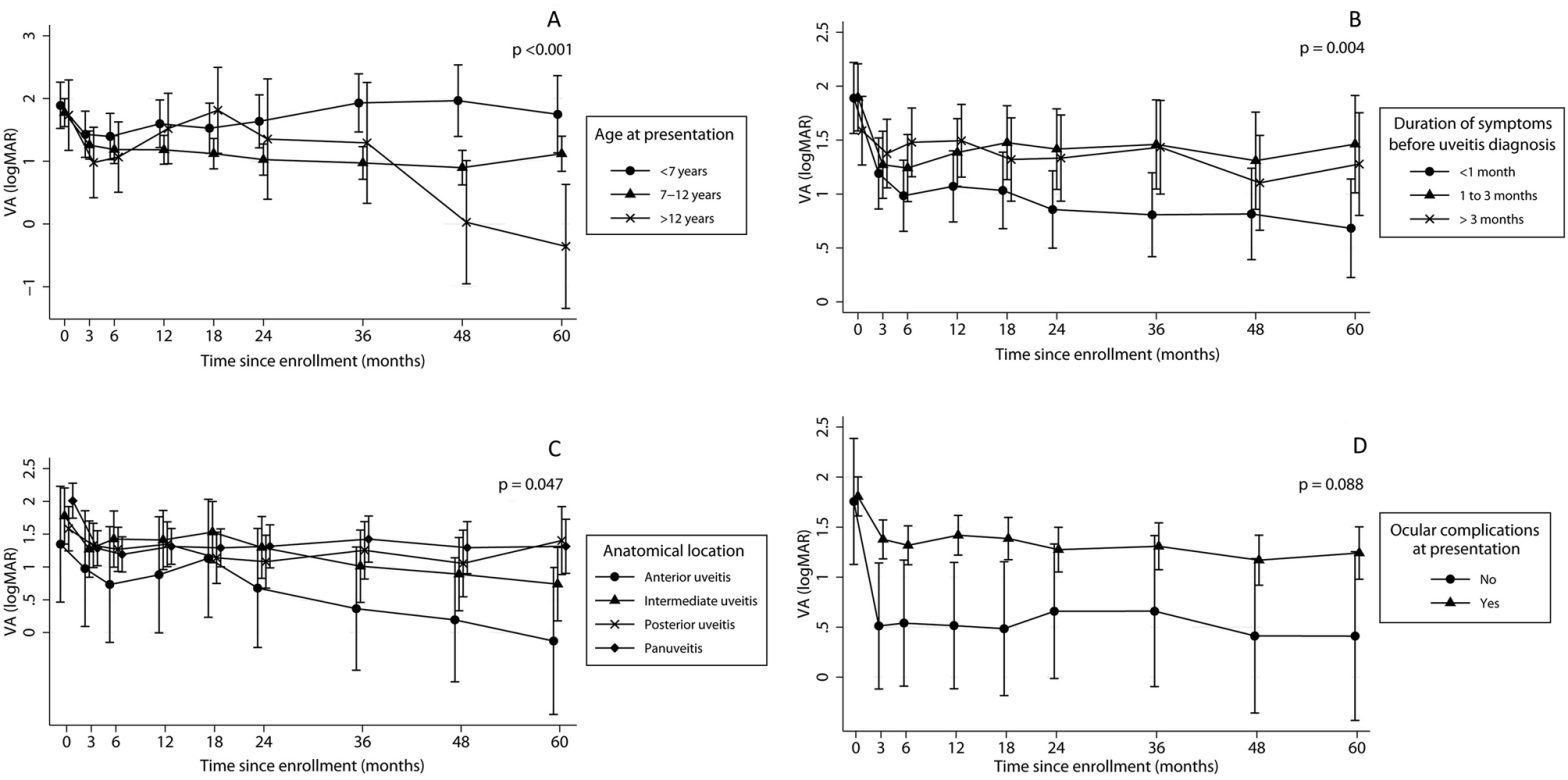
Multivariate mixed-effects linear regression results show the associations between predictive mean VA (logMAR) over 5 years after presentation and (**A**) age, (**B**) duration of symptoms before diagnosis, (**C**) anatomical location, and (**D**) ocular complications at presentation. P-values were derived using the joint Wald test over time from 0 to 60 months. *logMAR* logarithm of the minimum angle of resolution, *VA* visual acuity.

Mixed-effect linear regression analysis was performed to identify significant predictive factors associated with VA outcome over the 5-year follow-up. Only one eye was categorized as recurrent course of uveitis and could not be fit to the model. Therefore, this eye was excluded from the regression analyses. Hence, 56 eyes from 50 patients were analyzed for predictive factors of VA outcome. The variables initially included in the multivariate model were age at uveitis onset, duration of symptoms before uveitis diagnosis, laterality, course, anatomical location of uveitis, diagnosis of uveitis (infectious or non-infectious), and presence of any ocular complications at presentation. After model refinement, age at uveitis onset, duration of ocular symptoms before uveitis diagnosis, and anatomical location of uveitis remained significantly associated with VA outcome. The model, which was adjusted for repeated measures on patient and eye, demonstrated poorer VA outcome in eyes with preschool-age uveitis onset (P < 0.001). The mean VA in the preschool age group worsened after 2 years and returned to a level similar to the VA at presentation (Fig. 3A). Improvement in VA was more pronounced in eyes with < 1 month of ocular symptoms prior to uveitis diagnosis than in those with a longer duration (P = 0.004) (Fig. 3B). Additionally, eyes with anterior uveitis showed greater VA improvement than those with intermediate uveitis, posterior uveitis, or panuveitis (P = 0.047) (Fig. 3C). Eyes without ocular complications at presentation appeared to achieve greater VA improvement than those with ocular complications; however, the difference was not significant (P = 0.088) (Fig. 3D). The mean logMAR VA values at each timepoint for all variables included in the final multivariate mixed-effect linear regression model are presented in Supplementary Table S1.

## Discussion

This study showed that pediatric patients with uveitis who presented with SVI in at least one eye were mainly characterized as having unilateral disease, chronic course, and panuveitis. Most children were in the primary school age group, infectious uveitis was more common than uveitis associated with systemic inflammatory disease, and ocular complications at presentation were highly prevalent. On average, those eyes experienced significant VA improvement in the first 3 months after starting uveitis treatment, and these improvements were relatively well maintained over 5 years of follow-up. However, the degree of VA improvement was limited and remained at the SVI level. A more favorable VA outcome was observed in eyes with uveitis onset at school age or older, anterior uveitis, and a shorter duration of ocular symptoms before uveitis diagnosis.

As expected, approximately 95% of patients had uveitis involving the posterior segment. Uveitis at this anatomic location is typically more severe and is frequently associated with sight threatening ocular complications. Our findings support those of previous studies that have described a greater proportion of VA impairment at presentation among children with posterior uveitis and panuveitis^5,25,26^. In addition, the etiology of uveitis might contribute to the severity of VA loss. Infection, particularly ocular toxoplasmosis, has been described as the etiology associated with poor VA outcomes in earlier studies owing to the macular scar sequelae^5,8,13,27^. Ocular toxoplasmosis was also the most common uveitis diagnosis in the present study, with three out of five eyes with toxoplasmosis having consistently poor VA (≤ 20/200) through the final visit despite effective antiparasitic treatment. Furthermore, most children with ocular toxocariasis and diffuse unilateral subacute neuroretinitis also had a poor rate of VA recovery as they developed tractional RD and diffuse retinal atrophy later in the course of treatment, which permanently limited their vision.

In this cohort of children, infectious uveitis was more prevalent than non-infectious uveitis. Furthermore, JIA-associated uveitis, which accounts for most pediatric patients with uveitis in Western countries, is a rare entity in Thailand and in Southeast Asia in general. The prevalence of JIA-associated uveitis reported from this region ranges from 0 to 1.5%^12,28–33^. Our previous publication on the pattern of pediatric uveitis that included all pediatric patients from 2010 to 2020 had identified JIA-associated uveitis in only two of 118 (1.7%) children and none of them had presenting VA of ≤ 20/200^12^. Hence, there were no children with JIA-associated uveitis enrolled in the present study.

The long-term course of VA outcome in this study demonstrated that, on average, eyes with both infectious and non-infectious uveitis experienced VA improvement shortly after receiving uveitis treatment. Although these improvements were statistically significant compared with the baseline VA, the final VA remained within the SVI level. When evaluating the VA outcome at the last follow-up visit, with the follow-up time varying for each eye, we observed that approximately half of all affected eyes remained suffering SVI after treatment. These persistent poor VA outcomes were likely attributed to the presence of vision-threatening complications that potentially lead to permanent visual loss, such as RD, optic atrophy, glaucoma, or atrophic bulbi. Rhegmatogenous RD and tractional RD at any time were noted in seven and 13 eyes, respectively. Surgical repair for RD was performed in nine eyes. However, none of them achieved successful anatomical retinal reattachment, and all eyes had a poor VA of < 20/200 at final follow-up. The observation of VA improvement at 3 months with relatively well-maintained VA thereafter despite a large proportion of eyes developing new-onset ocular complication during follow-up was likely explained by the fact that some complications we reported were mild and did not significantly affect vision when managed appropriately, such as ocular hypertension, posterior synechiae, or optic disc/retinal neovascularization. Furthermore, some were treatable complications with potential for VA improvement, such as cataract, macular edema, and vitreous hemorrhage.

Regarding predictive factors of VA outcome, preschool age was associated with poorer outcomes than older ages. This was in accordance with the study performed by Ganesh et al., who reported poorer visual prognosis in children with uveitis who presented at a younger age with a longer duration of uveitis prior to referral to a tertiary care center^34^. Our results showed that among the seven eyes (seven patients) that subsequently developed atrophic bulbi related to uveitis, four belonged were preschool-aged. The poorer VA outcome of younger patients may be attributed to the greater severity of uveitis and the challenges related to medication compliance, which relies almost solely on parents. In addition, amblyopia may have the greatest impact on younger children, as their visual development is interrupted since early childhood. Although the treatable component of amblyopia was corrected, VA improvement was still limited. Anterior uveitis was an independent predictive factor associated with greater VA improvement in our study. This could be explained by the decreased likelihood of developing irreversible ocular complications, which limits VA recovery in eyes with anterior uveitis. The median number of complications per eye among eyes with anterior uveitis and those with uveitis involving the posterior segment was 1 (range, 0–5) and 4 (range, 0–9) (P = 0.169), respectively.

Our results support the findings that a longer duration of uveitis and delayed referral to a tertiary care center are associated with poorer VA outcomes^33–35^. We found that a long duration of uveitis of ≥ 1 month increased the likelihood of developing vision-threatening ocular complications at presentation, including RD, glaucoma, optic atrophy, and hypotony, compared to eyes with uveitis duration of ≤ 1 month (P = 0.003). This highlights the importance of early detection and prompt referral of children to uveitis subspecialty care when possible, as there is still potential for visual recovery even with severe vision loss at first presentation.

The limitations of this study include its small sample size and patients lost to follow-up over time, which is a reality in retrospective observational studies. The apparently stable VA from 6 months throughout 5 years may not completely reflect the outcome of the overall sample of patients. Heterogeneity of uveitis diagnoses, which were analyzed together in the final model, might have affected the results of associations of VA outcome since the natural course and/or severity of each specific uveitis diagnosis can intrinsically differ. In addition, our results may not be generalizable to other populations who live in different geographic areas with different climates, sociodemographic characteristics, and healthcare systems as there is a variability of types and etiologies of uveitis. Infectious uveitis was more frequently observed than non-infectious uveitis in the present study. The predictive factors included in our analysis models were all clinical characteristics at presentation, and there might be other time-dependent covariates, such as degree of intraocular inflammation during follow-up or effect of specific medication, that could be predictive of VA outcome but went unrecognized as time-updated data assessment was not performed. Despite these limitations, our study provided an overall long-term pattern of VA among children with uveitis who had severe visual loss at presentation and highlighted some presenting characteristics that could be predictive of visual improvement. This information would be helpful for the initial counseling of parents regarding the visual prognosis following treatment.

In conclusion, pediatric patients with uveitis in Thailand who presented with SVI were mainly characterized by chronic, posterior, or panuveitis and a high prevalence of ocular complications at presentation. Although these eyes experienced VA improvement in the first 3 months after starting uveitis treatment, and such improvement appeared to be stably maintained thereafter, the degree of VA improvement was limited and remained within the SVI level. The three most common causes of poor vision at final follow-up were RD, atrophic bulbi, and optic atrophy. Greater improvement in VA was observed in eyes with uveitis onset at school age or older, anterior uveitis, and a shorter duration of uveitis.

## Supplementary Information

Supplementary Table S1.

## Data Availability

The datasets generated during and/or analyzed during the current study are available from the corresponding author on reasonable request.
