# Supplementary material for: Long-term visual acuity outcome of pediatric uveitis patients presenting with severe visual impairment
Source: Sci Rep. 2023 Feb 20;13:2919. doi: 10.1038/s41598-023-29159-x (PMC9941571; doi:10.1038/s41598-023-29159-x)
Supplement: Supplementary file 1 — Supplementary Table S1. [file 41598_2023_29159_MOESM1_ESM.docx]

**Long-term Visual Acuity Outcome of Pediatric Uveitis Patients Presenting with Severe Visual Impairment**

Usanee Tungsattayathitthan, M.D.; Narisa Rattanalert, M.D.; Wantanee Sittivarakul, M.D*.

**Affiliation**

Department of Ophthalmology, Faculty of Medicine, Prince of Songkla University, Hat Yai, Songkhla, Thailand

***Corresponding Author:**

Wantanee Sittivarakul, M.D.

Department of Ophthalmology

Faculty of Medicine, Prince of Songkla University

15 Karnjanavanich Road, Hat Yai, Songkhla, Thailand 90110

Tel: +66 74 451380

Fax: +66 74 429619

Email: wantanee.s@psu.ac.th

ORCID iD: 0000-0003-1142-0345

**Supplementary Table S1.** Marginal effects of each variable adjusted for all other variables in the multivariate mixed-effects linear regression model.

| **Variable** | | **Mean logMAR VA (95% CI) at each time point** | | | | | | | | | **p-value**^a^ |
| --- | --- | --- | --- | --- | --- | --- | --- | --- | --- | --- | --- |
|  |  | **Baseline** | **3 months** | **6 months** | **12 months** | **18 months** | **24 months** | **36 months** | **48 months** | **60 months** |  |
| **Age group** | Preschool  (< 6 years) | 1.89  (1.52, 2.26) | 1.43  (1.06, 1.80) | 1.40  (1.03, 1.76) | 1.60  (1.22, 1.98) | 1.53  (1.13, 1.93) | 1.64  (1.21, 2.06) | 1.93  (1.47, 2.39) | 1.97  (1.40, 2.54) | 1.75  (1.13, 2.36) | <0.001 |
|  | Primary school  (7–12 years) | 1.78  (1.55, 1.99) | 1.26  (1.04, 1.48) | 1.19  (0.96, 1.41) | 1.18  (0.95, 1.41) | 1.12  (0.88, 1.36) | 1.03  (0.78, 1.27) | 0.97  (0.71, 1.23) | 0.90  (0.62, 1.17) | 1.12  (0.84, 1.40) |  |
|  | Secondary school  (> 13 years) | 1.74  (1.17, 2.30) | 0.98  (0.42, 1.54) | 1.07  (0.51, 1.63) | 1.52  (0.96, 2.08) | 1.81  (1.13, 2.50) | 1.35  (0.39, 2.31) | 1.29  (0.33, 2.26) | 0.03  (-0.95, 1.01) | -0.36  (-1.35, 0.63) |  |
| **Duration of symptom before diagnosis** | < 1 month | 1.89  (1.56, 2.22) | 1.19  (0.86, 1.52) | 0.98  (0.65, 1.31) | 1.07  (0.74, 1.40) | 1.03  (0.68, 1.39) | 0.86  (0.50, 1.21) | 0.81  (0.42, 1.20) | 0.82  (0.39, 1.24) | 0.68  (0.23, 1.14) | 0.004 |
|  | 1–3 months | 1.90  (1.58, 2.21) | 1.27  (0.96, 1.58) | 1.24  (0.93, 1.55) | 1.38  (1.07, 1.70) | 1.48  (1.13, 1.82) | 1.42  (1.04, 1.79) | 1.46  (1.05, 1.87) | 1.31  (0.86, 1.76) | 1.46  (1.01, 1.91) |  |
|  | > 3 months | 1.59  (1.27, 1.91) | 1.38  (1.06, 1.69) | 1.48  (1.16, 1.80) | 1.49  (1.16, 1.83) | 1.32  (0.93, 1.71) | 1.33  (0.93, 1.73) | 1.43  (1.00, 1.87) | 1.10  (0.66, 1.54) | 1.28  (0.80, 1.75) |  |
| **Anatomical classification** | Anterior uveitis | 1.35  (0.47, 2.23) | 0.98  (0.09, 1.86) | 0.73  (-0.15, 1.61) | 0.88  (-0.004, 1.77) | 1.13  (0.23, 2.03) | 0.68  (-0.23, 1.59) | 0.36  (-0.57, 1.30) | 0.19  (-0.75, 1.14) | -0.13  (-1.25, 0.99) | 0.047 |
|  | Intermediate uveitis | 1.78  (1.35, 2.20) | 1.27  (0.84, 1.70) | 1.42  (1.00, 1.85) | 1.41  (0.96, 1.86) | 1.53  (1.06, 2.00) | 1.30  (0.83, 1.77) | 1.01  (0.46, 1.56) | 0.89  (0.33, 1.45) | 0.74  (0.18, 1.30) |  |
|  | Posterior uveitis | 1.58  (1.25, 1.92) | 1.33  (0.99, 1.67) | 1.27  (0.93, 1.61) | 1.35  (1.01, 1.69) | 1.14  (0.75, 1.53) | 1.08  (0.68, 1.48) | 1.25  (0.82, 1.69) | 1.05  (0.55, 1.56) | 1.40  (0.88, 1.92) |  |
|  | Panuveitis | 2.01  (1.74, 2.28) | 1.28  (1.02, 1.55) | 1.19  (0.92, 1.46) | 1.31  (1.04, 1.59) | 1.29  (1.00, 1.58) | 1.31  (0.99, 1.64) | 1.42  (1.07, 1.78) | 1.29  (0.90, 1.69) | 1.31  (0.90, 1.73) |  |
| **Complications at presentation** | No | 1.76  (1.13, 2.39) | 0.51  (-0.12, 1.14) | 0.54  (-0.09, 1.17) | 0.52  (-0.12, 1.15) | 0.49  (-0.18, 1.15) | 0.66  (-0.01, 1.33) | 0.66  (-0.09, 1.42) | 0.41  (-0.36, 1.18) | 0.41  (-0.43, 1.25) | 0.088 |
|  | Yes | 1.81  (1.61, 2.00) | 1.38  (1.18, 1.57) | 1.32  (1.12, 1.51) | 1.42  (1.22, 1.62) | 1.39  (1.17, 1.60) | 1.27  (1.05, 1.50) | 1.31  (1.07, 1.54) | 1.17  (0.92, 1.42) | 1.24  (0.98, 1.50) |  |

CI = confidence interval; logMAR = logarithm of the minimum angle of resolution; VA = visual acuity.

^a^ p-value, joint Wald test over time from 0 to 60 months.
